# Awareness of obstetric fistula and its associated factors among women of reproductive age in sub-Saharan Africa

**DOI:** 10.1186/s41182-022-00443-2

**Published:** 2022-08-02

**Authors:** Eugene Budu, Bright Opoku Ahinkorah, Joshua Okyere, Abdul-Aziz Seidu, Richard Gyan Aboagye, Sanni Yaya

**Affiliations:** 1 Department of Population and Health, University of Cape Coast, Cape Coast, Ghana; 2 School of Public Health, Faculty of Health, University of Technology Sydney, Sydney, Australia; 3 Department of Estate Management, Takoradi Technical University, Takoradi, Ghana; 4 College of Public Health, Medical and Veterinary Sciences, James Cook University, Townsville, Australia; 5 Department of Family and Community Health, School of Public Health, University of Health and Allied Sciences, Ho, Ghana; 6 School of International Development and Global Studies, University of Ottawa, Ottawa, Canada; 7 The George Institute for Global Health, Imperial College London, London, UK

**Keywords:** Obstetric, Fistula, Global health, Sub-Saharan Africa, Demographic and Health Survey

## Abstract

**Background:**

Awareness about obstetric fistula and its concomitant factors is central to efforts to eliminate obstetric fistula in sub-Saharan Africa. We, therefore, assessed the magnitude of obstetric fistula awareness and its associated factors among women of reproductive age in sub-Saharan Africa.

**Methods:**

Data for the study were extracted from the most recent Demographic and Health Surveys of 14 countries in sub-Saharan Africa. We included 185,388 women aged 15–49 years in this study. Percentages were used to summarise the prevalence of obstetric fistula awareness across the 14 countries studied. We adopted a multivariable multilevel binary logistic regression to examine the factors associated with obstetric fistula awareness in sub-Saharan Africa. We presented the results of the regression analysis using adjusted odds ratios with their 95% confidence intervals. Statistical significance was set at *p* < 0.05.

**Results:**

The average prevalence of obstetric fistula awareness was 37.9%, ranging from 12.8% in Gambia to 63.9% in Uganda. Awareness of obstetric fistula was low among never married and cohabiting women compared to married women. Compared with women with parity 4 or more, those with no birth had the lowest odds of obstetric fistula awareness. The study also showed that obstetric fistula awareness was lower among women who were working, those who are not exposed to mass media, those in the poorest wealth category, those who have never had sex, and those in communities with low literacy level. The study however found that the odds of obstetric fistula awareness increased with age and education, and was higher in urban areas compared to rural areas. Women, who had ever terminated a pregnancy were more likely to be aware of obstetric fistula compared to those who had never terminated a pregnancy.

**Conclusion:**

The study demonstrated a low awareness of obstetric fistula among women in sub-Saharan Africa. Educative and sensitisation interventions should incorporate the factors identified in the present study during its implementation. To raise women’s awareness of obstetric fistula, there is the need for sub-Saharan African countries to consciously raise community literacy rate, increase access to mass media platforms and invest intensively in formal education for women.

## Background

Motherhood is an important milestone in the lives of most women. Yet, this phase of life which should be a bundle of joy and happiness tends to be often marred with some challenges. Some women either suffer complications during pregnancy and childbirth, thereby leading to the death the child or mother [1]. One of such birth-related injuries that exacerbate maternal morbidity and mortality is obstetric fistula (OBF) [2]. OBF is a medical condition in which there is an abnormal opening or connection between a woman’s genitals and the urinary tract or rectum [2, 3]. Usually, OBF is a characteristic of prolonged and obstructed labour, which is worsened by the unavailability of prompt medical care [4].

Although OBF occurs in both low-and middle-income and high income countries, the greatest burden of OBF are recorded in low-and middle-income  countries [4]. Evidence shows that each year, between 50,000 and 100,000 cases of OBF are reported worldwide [5]. The statistics are staggering for sub-Saharan Africa (SSA) as the incidence rate of OBF stands at 10 cases per 1000 births [4]. Generally, women who experience OBF are stigmatised and forced to live a life of misery, loneliness, and poverty [6]. Thus, underscoring the need to eliminate OBF. Recognising the need to eliminate OBF, the World Health Organization (WHO) has outlined strategies to prevent OBF. These strategies include promoting prompt access to obstetric care, delaying maternal age at first birth, and the elimination of harmful traditional procedures such as female genital mutilation (FGM) [6].

Awareness of OBF and its related factors is central to efforts to eliminate OBF in SSA. Earlier studies conducted in individual sub-Saharan countries have found varied responses to women’s awareness of OBF. For instance, in a study conducted in the Ebonyi State of Nigeria, it was revealed that nearly 58% of women were aware of OBF [1]. However, another study conducted in Ghana showed that 29% were aware of OBF, while 37 and 57% of women had poor knowledge and misconceptions about OBF, respectively [4]. Studies conducted in Ghana [4], Nigeria [1], and Ethiopia [7] have shown that maternal level of education, parity, place of birth, antenatal care attendance, maternal age, distance to a health facility, and caesarean section are the factors associated with the awareness of OBF.

Despite the high burden of OBF in SSA [8], there is a paucity of nationally representative studies that have assessed OBF awareness and its associated factors from a regional perspective. The existing studies in SSA investigating this phenomenon [1, 4, 7] approached it from a country-specific perspective. Hence, the awareness of OBF and its associated factors across SSA remains unclear. We, therefore, assessed the magnitude of OBF awareness among women of reproductive age in SSA. Additionally, the study determines the factors associated with OBF awareness in SSA. This study is critical to the attainment of Sustainable Development Goal (SDG) 3.1, which envisions to “reduce the global maternal mortality ratio to less than 70 per 100,000 live births by 2030” [9]. Assessing the awareness of OBF and its factors would allow women to take up the preventive strategies as postulated by the WHO. Also, information on awareness about OBF will alert health professionals and support organisations about the need for primary prevention through sensitisation, promotion of contraceptive use and appropriate birth spacing. Creating awareness on OBF among women in the reproductive age group haS a crucial role in reducing morbidity, mortality, and social stigma. Adequate information and awareness of the risk factors, causes and treatment options for OBF may help women to take appropriate steps to prevent OBF [1].

## Methods

### Data source and study design

Data for the study were extracted from the most recent Demographic and Health Surveys (DHS) of 14 countries in SSA. We pooled the data from the women’s recode files in each of the 14 countries. The DHS is a comparatively nationally representative survey conducted in over 85 low- and-middle-income countries worldwide [10]. The DHS are nationally representative household surveys that provide data for a wide range of monitoring and impact evaluation indicators in the areas of population, health, and nutrition. All survey data are presented both nationally and by sub-national reporting area. The sample is generally representative: at the national level, at the residence level (urban–rural), and at the regional level (departments, states).

The DHS develops data processing guidelines to assist DHS staff and local collaborators in developing data processing procedures. While the quality of the data is determined mainly by the quality of the fieldwork, following appropriate steps can enhance it significantly during data processing. Data entry and editing for inconsistencies are major steps in this process, as is the handling of missing data. In this study, missing data were handled through list-wise deletion. DHS employed a descriptive cross-sectional design. Respondents for the survey were recruited using a two-stage cluster sampling method. Detailed sampling technique has been highlighted in the literature [11]. Standardised structured questionnaires were used to collect data from the respondents on health indicators, including place and mode of delivery. To gather high-quality data for the DHS, many different quality control procedures are used. By using the same variables and metrics across the board, for instance, consistency is preserved. However, nations are free to add particular variables that are relevant to their situation. The survey team consists of trainees who have received training in conventional DHS practises, such as general interviewing skills, conducting household-level interviews, reviewing each question, and performing mock interviews between participants. Depending on the nation's official language, DHS in sub-Saharan Africa are typically done in English, French, or Portuguese. The definitive questionnaires are first written in the official language in the particular country, and then translated into the primary local languages at the various data collection stations to make sure participants understood the questions being asked [10, 11]. We included 185,388 women in this study (Table 1). The datasets used are freely available at https://dhsprogram.com/data/available-datasets.cfm. This manuscript was drafted with reference to the Strengthening the Reporting of Observational Studies in Epidemiology (STROBE) statement guidelines [12].

**Table 1:** Description of study sample

Country	Survey year	Weighted N	Weighted %
1.Burkina Faso	2010	16,511	8.9
2.Congo	2013	10,542	5.7
3.Cameroon	2018	13,492	7.3
4.Ethiopia	2016	15,139	8.2
5.Gambia	2019–20	11,804	6.4
6.Guinea	2014	10,108	5.4
7.Kenya	2014	14,301	7.7
8.Mali	2018	10,358	5.6
9.Nigeria	2018	18,036	9.7
10.Niger	2012	11,026	6.0
11.Senegal	2010–11	15,638	8.4
12.Chad	2014–15	11,031	5.9
13.Togo	2013–14	9336	5.0
14.Uganda	2016	18,065	9.7
All countries		185,388	100.0

### Variables

#### Outcome variable

The outcome variable of this study was women’s awareness of OBF. The variable measures the extent to which women are aware of OBF. This variable was derived from the question “have you ever heard about fistula?” Responses to this question were categorised into “No” and “Yes”. The variable was dichotomised into 1 = “Yes, ever heard of fistula” and 0 = “No, never heard of fistula”. Studies that used the DHS dataset employed similar coding [13–15].

#### Explanatory variables

The explanatory variables considered in this study were selected based on their association with OBF awareness [13–15] and also their availability in the DHS dataset. Thirteen (13) variables were included in the study as explanatory variables. These variables were grouped as individual and contextual level factors. The individual level factors comprised mother’s age, educational level, marital status, employment status, parity, wealth index, exposure to mass media, sexual activity, pregnancy status, and pregnancy termination. For the contextual factors, we included type of place of residence, community literacy level, and community socioeconomic status. The categories of each of the variables are shown in Table 2.

**Table 2:** Distribution of awareness of among women in sub-Saharan Africa across the explanatory variables (n = 185,388)

Variables	Weighted N	Weighted %	Awareness of OBF	p-value
Age				< 0.001
15–19	37,214	20.1	24.6	
20–24	33,536	18.1	37.2	
25–29	33,881	18.3	40.3	
30–34	27,296	14.7	42.4	
35–39	22,943	12.4	42.9	
40–44	16,855	9.1	44.5	
45–49	13,661	7.4	45.1	
Marital status				< 0.001
Not married	43,807	23.6	28.4	
Married	110,777	59.7	40.8	
Cohabiting	16,816	9.1	40.3	
Widowed	4251	2.3	43.1	
Divorced	9737	5.3	42.6	
Parity				< 0.001
No birth	48,537	26.2	29.1	
One birth	24,321	13.1	37.2	
Two births	22,795	12.3	40.6	
Three births	20,639	11.1	41.1	
Four or more births	69,096	37.3	42.6	
Employment status				< 0.001
Not working	64,844	35.0	30.4	
Working	120,544	65.0	42.0	
Exposure to mass media				< 0.001
Not exposed	52,501	28.3	30.5	
Exposed	132,887	71.7	40.9	
Wealth index				< 0.001
Poorest	31,961	17.2	33.0	
Poorer	34,413	18.6	34.6	
Middle	35,984	19.4	34.5	
Richer	38,806	20.9	36.8	
Richest	44,224	23.9	47.9	
Level of education				< 0.001
No education	77,603	41.9	34.3	
Primary	48,808	26.3	40.0	
Secondary	49,095	26.5	37.2	
Higher	9882	5.3	59.9	
Sexual activity				< 0.001
Never had sex	28,149	15.2	24.3	
Ever had sex	157,239	84.8	40.4	
Currently pregnant				0.137
Not pregnant	167,997	90.6	37.8	
Pregnant	17,391	9.4	39.0	
Ever terminated a pregnancy				< 0.001
No	158,859	85.7	37.0	
Yes	26,589	14.3	43.7	
Type of place of residence				< 0.001
Urban	72,915	39.3	38.8	
Rural	112,473	60.7	37.4	
Community literacy level				< 0.001
Low	55,954	30.2	34.4	
Medium	62,121	33.5	36.3	
High	67,313	36.3	42.4	
Community socio-economic status				< 0.001
Low	81,982	44.2	35.1	
Moderate	34,857	18.8	36.6	
High	68,549	39.0	42.0	

P-values are generated from Chi-square test

### Statistical analyses

Data for the study were analysed using Stata version 16. First, a bar chart was used to show the prevalence of the awareness of OBF across the 14 countries. Next, the weighted frequencies and percentages for the explanatory variables were presented. Later, we presented the bivariate results on the distribution of awareness of OBF across the explanatory variables using Chi-square test of independence (Table 2). After this, we checked for multicollinearity among the explanatory variables using the variance inflation factor (VIF) and the results showed no evidence of high collinearity (Maximum VIF = 2.95, Minimum VIF = 1.06 and Mean VIF = 1.84). Finally, a four modelling multivariable multilevel binary logistic regression (Model 0-III) was employed to examine the factors associated with OBF awareness. Model 0 was an empty model with no outcome or explanatory variable. Model I had only the individual-level variables. Model II had only the contextual variables while Model III, which was considered as the complete model had both the individual and contextual level factors. The results of the fixed effects of the model were presented as adjusted odds ratio (aOR) while the random effects were assessed with Intra-Cluster Correlation (ICC). Model comparison was done using the log-likelihood ratio (LLR) and Akaike’s Information Criterion (AIC) tests.  All frequency distributions were weighted (v005/1000000) while the survey command (svy) in Stata was used to adjust for the complex sampling structure of the data in the regression analyses.

### Ethical consideration

In this study, ethical clearance was not sought due to the public availability of the DHS dataset. The datasets were obtained from the MEASUREDHS after registration and approval were given for its usage. All the ethical guidelines concerning the use of secondary datasets in the publication were strictly adhered to. Detailed information about the DHS data usage and ethical standards are available at http://goo.gl/ny8T6X.

## Results

**Fig. 1:**
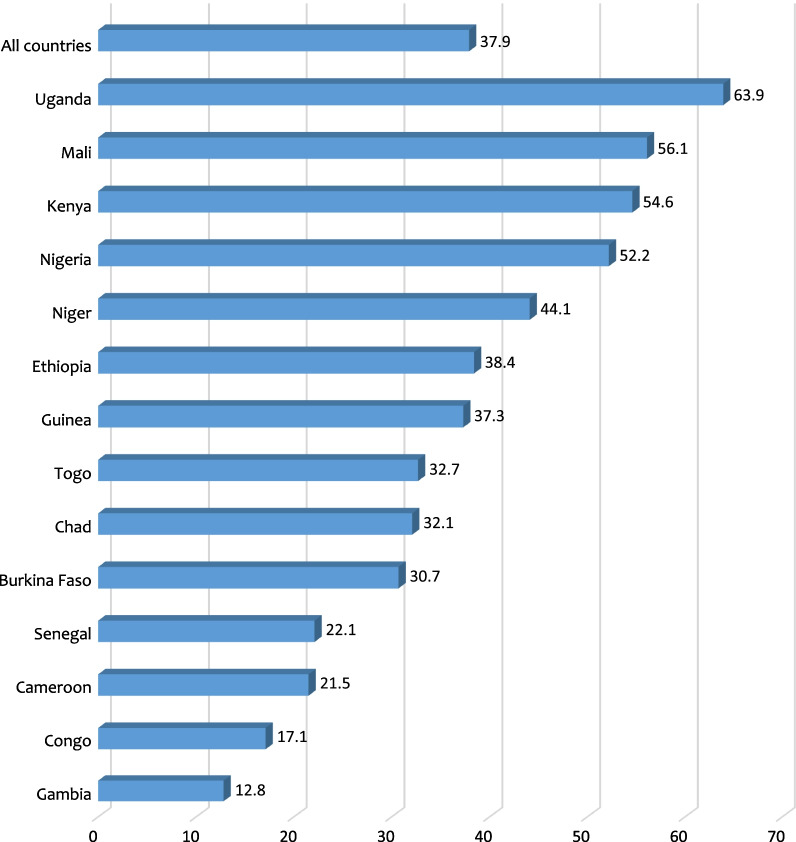
Prevalence of awareness of obstetric fistula among women in sub-Saharan Africa

Figure 1 shows the prevalence of awareness of OBF among women in SSA. The average prevalence was 37.9%, ranging from 12.8% in Gambia to 63.9% in Uganda.

### Distribution of awareness of obstetric fistula among women in sub-Saharan Africa across the explanatory variables

Table 2 shows the distribution of awareness of OBF among women in SSA across the explanatory variables. Awareness of OBF was highest among those aged 45–49 years (45.1%), widowed (43.1%), those with four or more births (42.6%), those working (42.0%), those exposed to mass media (40.9%), the richest (47.9%) and those with higher level of education (59.9%). Except pregnancy status, all the explanatory variables showed statistically significant association with OBF awareness.

### Fixed effect (measures of association) results  on the factors associated with  obstetric fistula awareness among women in sub-Saharan Africa

Table 3 shows the results of the multilevel analysis on the predictors of OBF awareness. With marital status, those who were not married (aOR = 0.80;95% CI = 0.77–0.84) or cohabiting (aOR = 0.91,95% CI = 0.87–0.95) had lower odds of OBF awareness compared with married women. In terms of parity, it was found that compared with those with parity 4 or more, women with no birth (aOR = 0.82;95% CI = 0.78–0.86) had the lowest odds of OBF awareness. The study also showed that fistula awareness was lower among those not working (aOR = 0.75; 95% CI = 0.73–0.77), those who are not exposed to mass media (aOR = 0.68; 95% CI = 0.66–0.70), those in the poorest wealth category(aOR = 0.63; 95% CI = 0.60–0.67), those who have never had sex (aOR = 0.86; 95% CI = 0.81–0.90), and those in communities with low literacy level (aOR = 0.82; 95% CI = 0.79–0.85) compared with those who are working, exposed to the mass media, those in richest wealth quintile, those who have ever had sex, and those in communities with high literacy level. The odds of fistula awareness increased with age, with the highest among those aged 45–49 [aOR = 1.90; 95% CI = 1.79–2.01). With level of education, those with higher education (aOR = 2.45 (2.30–2.60) had the highest odds of fistula awareness. With place of residence, those in the urban area (1.07; 95% CI = 1.03–1.11) were more likely to be aware of OBF compared to those in rural areas. Those who have ever terminated a pregnancy were more likely (aOR = 1.26; 95% CI = 1.22–1.30) to be aware of OBF compared to those who have never terminated a pregnancy. Compared to women from Uganda, those from the remaining 13 countries had lower odds of OBF awareness.

**Table 3:** Fixed effect (measures of association) results on the factors associated with awareness of obstetric fistula among women in sub-Saharan Africa

Variables	Null model	Model I aOR [95% CI]	Model II aOR [95% CI]	Model III aOR [95% CI]
Age				
15–19		Reference (1.0)	Reference (1.0)	Reference (1.0)
20–24		1.34*** (129–1.40)	1.36*** (1.31–1.41)	1.44*** (1.39–1.51)
25–29		1.39*** (1.31–1.43)	1.40*** (1.34–1.46)	1.57*** (1.50–1.65)
30–34		1.39*** (1.32–1.46)	1.42*** (1.36–1.49)	1.64*** (1.56–1.73)
35–39		1.44*** (1.37–1.52)	1.48*** (1.40–1.55)	1.75*** (1.66–1.85)
40–44		1.47*** (1.39–1.55)	1.50*** (1.42–1.59)	1.80*** (1.70–1.91)
45–49		1.54*** (1.45–1.63)	1.57*** (1.48–1.66)	1.90*** (1.79–2.01)
Marital status				
Married		Reference (1.0)	Reference (1.0)	Reference (1.0)
Not married		0.76*** (0.73–0.80)	0.77*** (0.73–0.80)	0.80*** (0.77–0.84)
Cohabiting		0.83*** (0.80–0.86)	0.82*** (0.79–0.85)	0.91*** (0.87–0.95)
Widowed		0.97 (0.91–1.04)	0.98 (0.92–1.05)	0.93* (0.87–1.00)
Divorced		0.98 (0.93–1.02)	0.99 (0.95–1.04)	0.96 (0.91–1.00)
Parity				
No birth		0.76*** (0.73–0.80)	0.77*** (0.74–0.81)	0.82*** (0.78–0.86)
One birth		0.80*** (0.76–0.83)	0.81*** (0.78–0.84)	0.88*** (0.85–0.92)
Two births		0.86*** (0.83–0.89)	0.87*** (0.84–0.90)	0.92*** (0.89–0.96)
Three births		0.87*** (0.83–0.90)	0.87*** (0.84–0.90)	0.92*** (0.88–0.95)
Four or more births		Reference (1.0)	Reference (1.0)	Reference (1.0)
Employment status				
Not working		0.76*** (0.74–0.78)	0.77*** (0.75–0.78)	0.75*** (0.73–0.77)
Working		Reference (1.0)	Reference (1.0)	Reference (1.0)
Exposure to mass media				
Not exposed		0.73*** (0.72–0.75)	0.73*** (0.71–0.74)	0.68*** (0.66–0.70)
Exposed		Reference (1.0)	Reference (1.0)	Reference (1.0)
Wealth index				
Poorest		0.48*** (0.46–0.49)	0.43*** (0.41–0.45)	0.63*** (0.60–0.67)
Poorer		0.54*** (0.52–0.56)	0.48*** (0.46–0.50)	0.70*** (0.66–0.73)
Middle		0.54*** (0.52–0.56)	0.49*** (0.47–0.51)	0.67*** (0.64–0.70)
Richer		0.60*** (0.58–0.62)	0.57*** (0.55–0.59)	0.69*** (0.67–0.72)
Richest		Reference (1.0)	Reference (1.0)	Reference (1.0)
Level of education				
No education		Reference (1.0)	Reference (1.0)	Reference (1.0)
Primary		1.37*** (1.33–1.40)	1.36*** (1.32–1.40)	1.20*** (1.16–1.23)
Secondary		1.28*** (1.24–1.32)	1.28*** (1.24–1.32)	1.52*** (1.47–1.58)
Higher		2.50*** (2.37–2.64)	2.49*** (2.35–2.63)	2.45*** (2.30–2.60)
Sexual activity				
Never had sex		0.82*** (0.78–0.86)	0.83*** (0.79–0.87)	0.86*** (0.81–0.90)
Ever had sex		Reference (1.0)	Reference (1.0)	Reference (1.0)
Ever terminated a pregnancy				
No		Reference (1.0)	Reference (1.0)	Reference (1.0)
Yes		1.08*** (1.05–1.11)	1.08*** (1.05–1.11)	1.26*** (1.22–1.30)
Type of place of residence				
Urban			0.70*** (0.68–0.72)	1.07*** (1.03–1.11)
Rural			Reference (1.0)	Reference (1.0)
Community literacy level				
Low			0.81*** (0.78–0.84)	0.82*** (0.79–0.85)
Medium			0.85*** (0.82–0.88)	0.85*** (0.83–0.88)
High			Reference (1.0)	Reference (1.0)
Community socio-economic status				
Low			Reference (1.0)	Reference (1.0)
Moderate			0.99 (0.96–1.02)	0.87*** (0.84–0.90)
High			1.01 (0.97–1.05)	0.90*** (0.86–0.90)
Country				
Burkina Faso				0.27*** (0.26–0.29)
Congo				0.10*** (0.09–0.11)
Cameroon				0.14*** (0.13–0.15)
Ethiopia				0.57*** (0.54–0.60)
Gambia				0.08*** (0.07–0.08)
Guinea				0.39*** (0.37–0.41)
Kenya				0.83*** (0.37–0.41)
Mali				0.80*** (0.75–0.85)
Nigeria				0.67*** (0.63–0.71)
Niger				0.61*** (0.58–0.65)
Senegal				0.17*** (0.16–0.18)
Chad				0.37*** (0.35–0.39)
Togo				0.24*** (0.23–0.26)
Uganda				Reference (1.0)

***p < 0001

### Random effect (measures of variation) results on the factors associated with obstetric fistula awareness among women in sub-Saharan Africa

The ICC value for the null model (0.0681835) demonstrates that 6.8% of the variation in awareness of OBF was attributed to the variation between clusters (Table 4). This variation between clusters then increased to 8.3% in Model I that is individual-level only model (ICC=0.083465). In the contextual level model (Model II), the ICC reduced to 7.1% (ICC=0.0712482), while in the complete model, it increased again to 11.8% (ICC=0.1182673) (Model III). This reiterates that the variations in the awareness of OBF are attributed to the individual and contextual level factors. Model III which is the complete model with individual and contextual level factors had the  lowest Akaike Information Criterion (AIC) compared to the other models affirming the goodness of fit.

**Table 4:** Random effects (measures of variations) results on the factors associated with obstetric fistula awareness among women in sub-Saharan Africa

Random effect	Null model	Model I	Model II	Model III
PSU variance	0.24 (0.21–0.27)	0.30 (0.27–0.34)	0.25 (0.22–0.28)	0.44 (0.39–0.49)
ICC	0.0681835	0.083465	0.0712482	0.1182673
LR Test	3859.94 (X < 0.0001)	3481.0 (X < 0.0001)	3583.74 (X < 0.0001)	3732.62 (X < 0.001)
Wald Chi-square	Ref.	10,722.35***	2499.27***	22,811.54***
Model fitness				
Log-likelihood	− 12.0656.25	− 114,693.28	− 119,392.03	− 106,160.12
AIC	241,316.5	229,440.6	238,798.1	212,410.2
N	185,388	185,388	185,388	185,388
Number of groups	1612	1612	1612	1612

***p < 0001

## Discussion

Eliminating OBF is critical to the attainment of SDG target 3.1. This study assessed the magnitude of and factors associated with OBF awareness among women of reproductive age in SSA. Overall, the prevalence of OBF awareness across the 14 SSA countries included in this study was 37.9%. The estimated prevalence is less than what has been reported in individual SSA countries such as Ghana [16], Ethiopia [17], and Nigeria [1]. A plausible justification for this finding could be the heterogeneity in the study period, study design, and sample size. For instance, the studies conducted in Nigeria [1] and Ethiopia [17] were based only on the sample from those countries. Their sample cannot match that of our studies that included samples from 14 sub-Saharan African countries. Hence, the difference in sample size could account for the difference between our estimated magnitude and that of related studies in SSA. The findings also show that few women across SSA are aware of OBF. This may have dire consequences on sub-Saharan African countries’ quest to eliminate OBF as more women are likely to stay at home rather than seek treatment for OBF [7]. Hence, underscoring the need to strengthen public health interventions to raise awareness about OBF. Nevertheless, other proven alternatives that have significant impact in tackling the issue OBF include promoting prompt access to emergency obstetric and neonatal care, delaying maternal age at first birth, encouraging the use of contraception and birth spacing, and the elimination of harmful traditional procedures [6, 18]

Also, the study revealed that the prevalence of OBF awareness was not homogeneous across the 14 sub-Saharan African countries. Women in Uganda (63.9%) had the highest awareness of OBF compared to 12.8% in Gambia. It is uncertain why these differences exist. However, we postulate that this could be due to differences in the magnitude of OBF awareness programmes in each country. For instance, Kaji et al. [19] assert that in Uganda, the government often provides communities with information about repair camps by radio and through village health teams. This action has the tendency to significantly increase Ugandan women’s awareness level. Thus, emphasising the need for country-specific actions to increase women’s awareness of OBF.

In relation to the factors associated with OBF awareness, the results indicate that age was a significant factor. The likelihood of being aware of OBF increased significantly with increasing age. Thus, older women (i.e. older than 19 years) are more likely to be aware of OBF than younger women. This finding mirrors that of earlier studies conducted in Ethiopia [7] and Uganda [20]. All things being equal, it is expected that the older a woman gets, the more experience she would have with birth and its concomitant issues. As such, women older than 19 years are more likely to have undergone some counselling and had access to maternal health education opportunities that might have shaped their awareness about OBF [7].

Level of education also emerged significantly in predicting women’s awareness about OBF. This is consistent with several studies [7, 8, 17, 21] that have found that women with higher education are more likely to be aware of OBF compared to those with no formal education. For example, the study is substantiated by a study from Ethiopia [17] that showed that women with higher education were two times more likely to be aware of OBF as compared to women with no formal education. This may probably be due to the fact that formal education empowers women to make healthcare decisions such as seeking obstetric counselling and attending maternal health education forums which increases their awareness of OBF. Closely related to this finding was the observation that communities with low literacy rates were less likely to be aware of OBF when compared to communities with high literacy rates.

Compared to women who were exposed to mass media, women who were not exposed to mass media were less likely to be aware of OBF. The result corroborates the findings of previous studies as evidenced in Nigeria [15] and Ethiopia [7]. Presumably, the findings could be due to the fact that mass media is a key conduit for disseminating information including information about OBF, its signs and symptoms as well as information about where to access treatment. The findings of this showed that compared to women in rural residences, women in urban residences were more likely to be aware of OBF. The findings align with Morhason-Bello et al.’s [15] study in Nigeria that found the awareness of OBF to be higher among women in rural areas relative to women in urban areas.

Our study reveals a direct association between parity and women’s awareness of OBF. The lower the parity of the woman, the lower their awareness of OBF. The result is consistent with that of Asefa et al. [17] that found parity to be significantly associated with OBF awareness. A plausible explanation could be that higher parity comes with greater experience with obstetric and maternal education. Each childbirth offers women the opportunity to receive information about obstetric complications including OBF which raises their awareness level. Also, compared to married women, those who were unmarried or cohabiting were less likely to have OBF awareness.

The result also revealed that there is a strong significant association between pregnancy termination and OBF awareness. Similar finding was reported by Aleminew et al. [7]. This observation could be justified from the point that seeking healthcare services such as pregnancy termination services presents an opportunity for women to be exposed to health education and promotion messages, which may increase their awareness level of OBF. Our study also shows that compared to women who had ever had sex, those who had never had sex were less likely to be aware of OBF. Wealth index was significantly associated with OBF awareness. Women who belonged to a poorer wealth index were less likely to be aware of OBF as compared to those in a higher wealth index. Related studies from Ethiopia [7] and Nigeria [15] have all found a significant association between wealth index and women’s awareness of OBF. Women in high wealth index tend to have greater access to health facilities and institutional birth deliveries [22]. This comparative advantage that they enjoy as a result of their wealth index provides them the opportunity to access health information including information on OBF, thereby raising their awareness level.

## Strengths and limitations

By far, our literature review shows that this study is the first to assess the magnitude of and factors associated with OBF awareness based on nationally representative datasets of multiple countries in SSA. This is a significant contribution to existing literature. Nevertheless, there are some limitations worth discussing. The cross-sectional nature of the secondary data used does not allow us to make causal inferences to the factors associated with OBF awareness. Also, the DHS does not disaggregate questions to show the specific type of fistula (i.e. vesicovaginal fistula, urethrovaginal fistula, or rectovaginal fistula) that women are aware of. In the future, the DHS dataset could consider this disaggregation. We acknowledge that mixing the data can affect between country comparisons due to the different survey years. Finally, due to the large sample size, even the slightest shift becomes statistically significant with a dataset of this magnitude. The interpretation of the findings should be done taking into consideration the effect of the large sample size.

## Conclusion

We conclude that OBF awareness is low across SSA. It is evident from the study that age, level of education, wealth index, pregnancy termination, parity, marital status, community literacy rate, type of place of residence, and exposure to mass media were significant factors associated with OBF awareness. Educative and sensitisation interventions should incorporate the factors identified in the present study during their implementation. To raise women’s awareness of OBF, there is the need for countries in SSA to consciously raise community literacy rate, increase access to mass media platforms and invest intensively in formal education for women.

## Data Availability

Data are available in a public via the measuredhs website at https://dhsprogram.com/data/available-datasets.cfm
